# Application of satellite precipitation data to analyse and model arbovirus activity in the tropics

**DOI:** 10.1186/1476-072X-10-8

**Published:** 2011-01-22

**Authors:** Grit Schuster, Elizabeth E Ebert, Mark A Stevenson, Robert J Corner, Cheryl A Johansen

**Affiliations:** 1 Department of Spatial Sciences, Curtin University, GPO Box U1987, Perth, Western Australia 6845, Australia; 2 Weather and Environmental Prediction Centre for Australian Weather and Climate Research (CAWCR), Bureau of Meteorology, GPO Box 1289, Melbourne, Victoria 3001, Australia; 3 EpiCentre, Private Bag 11 222, Massey University, Palmerston North, New Zealand; 4 Arbovirus Surveillance and Research Laboratory, School of Biomedical, Biomolecular and Chemical Sciences, The University of Western Australia, Nedlands, Western Australia 6009, Australia

## Abstract

**Background:**

Murray Valley encephalitis virus (MVEV) is a mosquito-borne Flavivirus (Flaviviridae: *Flavivirus*) which is closely related to Japanese encephalitis virus, West Nile virus and St. Louis encephalitis virus. MVEV is enzootic in northern Australia and Papua New Guinea and epizootic in other parts of Australia. Activity of MVEV in Western Australia (WA) is monitored by detection of seroconversions in flocks of sentinel chickens at selected sample sites throughout WA.

Rainfall is a major environmental factor influencing MVEV activity. Utilising data on rainfall and seroconversions, statistical relationships between MVEV occurrence and rainfall can be determined. These relationships can be used to predict MVEV activity which, in turn, provides the general public with important information about disease transmission risk. Since ground measurements of rainfall are sparse and irregularly distributed, especially in north WA where rainfall is spatially and temporally highly variable, alternative data sources such as remote sensing (RS) data represent an attractive alternative to ground measurements. However, a number of competing alternatives are available and careful evaluation is essential to determine the most appropriate product for a given problem.

**Results:**

The Tropical Rainfall Measurement Mission (TRMM) Multi-satellite Precipitation Analysis (TMPA) 3B42 product was chosen from a range of RS rainfall products to develop rainfall-based predictor variables and build logistic regression models for the prediction of MVEV activity in the Kimberley and Pilbara regions of WA. Two models employing monthly time-lagged rainfall variables showed the strongest discriminatory ability of 0.74 and 0.80 as measured by the Receiver Operating Characteristics area under the curve (ROC AUC).

**Conclusions:**

TMPA data provide a state-of-the-art data source for the development of rainfall-based predictive models for Flavivirus activity in tropical WA. Compared to ground measurements these data have the advantage of being collected spatially regularly, irrespective of remoteness. We found that increases in monthly rainfall and monthly number of days above average rainfall increased the risk of MVEV activity in the Pilbara at a time-lag of two months. Increases in monthly rainfall and monthly number of days above average rainfall increased the risk of MVEV activity in the Kimberley at a lag of three months.

## Introduction

Murray Valley encephalitis virus (MVEV; Flaviviridae:*Flavivirus*) is a mosquito-borne arbovirus endemic to northern Australia and Papua New Guinea. MVEV virus can cause fatal disease in humans. While the fatality rate lies at 25%, 25-50% of people who develop clinical symptoms are permanently affected due to neurological damage [1].

**Figure 1:**
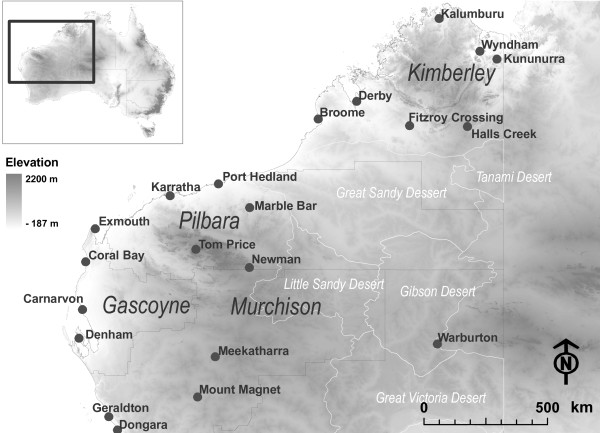
**Main areas of north and interior Western Australia**.

Small outbreaks of MVEV occur every few years throughout Australia, usually at the end of the wet season, between February and July [2]. The most recent Australia-wide outbreak was in 1974 [3]. During the wet season of 1999-2000, when record rainfall was recorded in the north of Australia, a very rapid and unusual spread of MVEV from the north to the south of WA was observed. Activity of MVEV occurred in epizootic regions such as the Gascoyne and the Murchison (Figure 1) and was detected 315 km north of metropolitan Perth [4] in late April 2000, representing a serious public health risk.

The MVEV transmission cycle is similar to the transmission cycles of other closely related medically important flaviviruses including Japanese encephalitis virus (JEV), West Nile virus (WNV) and St. Louis encephalitis virus (SLEV). *Culex annulirostris *mosquitoes are the principle vector and various species of migratory waterbirds, particularly of the Order *Ciconiiformes *are the major hosts [3]. As with other arboviruses, the spread of MVEV is linked to the abundance and distribution of vector and host populations which strongly depend on the availability and the conditions of suitable habitat. Habitat is influenced by multiple environmental factors such as rainfall and surface water, surface and air temperature, as well as vegetation type and distribution [5-7]. These influences are spatially and temporally highly variable.

**Figure 2:**
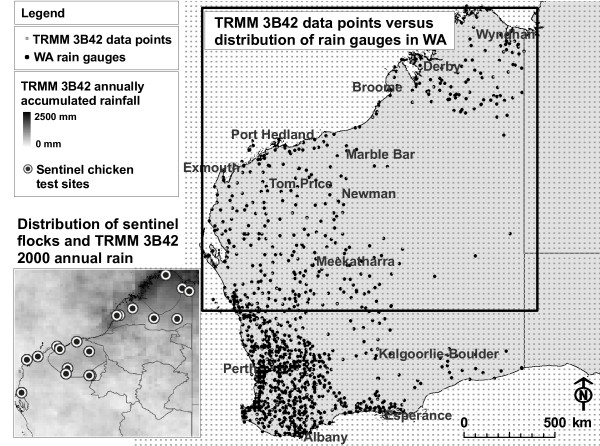
**Distribution of BoM rain gauges, TRMM 3B42 data points and serological sample sites**. Rain gauge coverage is sparse and irregular, especially in the north of WA. In many areas neighbouring stations are located within a radius of 100 or more kilometres. Small-scale rainfall might not be detected. The subset on the bottom left side shows TRMM 3B42 accumulated rainfall for 2000 and the sentinel chicken serological sample sites in WA. The final dataset used in this study comprises a total of nine sites in the Kimberley and eleven sites in the Pilbara. In some cases multiple sites are located within the same town.

Flavivirus activity in WA is monitored through the Western Australian Arbovirus Surveillance and Research Program [8]. The program incorporates flavivirus antibody detection in sentinel chicken sera. Serological data are collected at fortnightly to monthly intervals at approximately 30 test sites at the main populated centers throughout WA (Figure 2) using sentinel chickens [8-10]. One sentinel chicken flock ideally consists of twelve birds. However, in practice the numbers fluctuate between zero and twelve. The program has been in place since the 1980s, and provides information of virus presence or absence in the sentinel chickens at single point locations throughout WA. Similar programs monitoring other medically important arboviruses such as WNV and SLEV exist in the United States [11-15].

The data gathered by the program are subject to temporal and particularly spatial limitations, as virus activity is monitored at a limited number of locations. Due to the time and cost intensiveness this method of surveillance is not practicable in remote and inaccessible areas. The work reported here aims to enhance the current arbovirus surveillance system and to overcome limitations. By employing a combination of the ground based serological data and RS derived rainfall data, existing spatio-temporal relationships between arbovirus occurrence and rainfall related environmental conditions can be analysed and used to model MVEV occurrence risks.

In this paper, we are dealing exclusively with rainfall since it is one of the principal environmental factors influencing spatio-temporal vector and host dynamics and hence MVEV occurrence [3,16,17]. A basic prerequisite to overcoming some of the spatial limitations of the current surveillance is the utilisation of spatially coherent data. Rain gauges in WA are sparse and irregularly distributed, particularly in the northern areas of the state (Figure 2). Interpolation of rain-surfaces from very sparse datasets can lead to large errors, especially for arid regions of WA where rainfall is highly variable (Stafford Smith and Morton, 1990 [18] cited in Roshier et al. [19]). Weymouth et al. [20] illustrated a strong increase in interpolation error with decreasing density of the precipitation ground measurements in Australia. Further concerns about weather station data quality and also quality control as provided by the BoM, are incompleteness and inaccuracy of data records due to instrument failure, irregular calibration or absence of the observer. In contrast, RS data are collected repeatedly and automatically [21] and provide spatially regular information with a complete area-wide coverage, even in areas that are remote and difficult to access. Furthermore, many RS data products are available free of charge.

Satellite precipitation data are operationally derived from cloud properties [22]. The quality of this data depends on the measurement technique, the environmental conditions at the time of recording, and the algorithm used to derive geophysical data from the signal detected by a RS instrument. Furthermore, the performance of different data products is strongly related to the observed precipitation regime. To understand the accuracy and limitations of different data products and to determine those data most suitable for our study, evaluation and validation of the data are essential.

The International Precipitation Working Group (IPWG) hosts several projects dealing with the validation and intercomparison of different satellite precipitation and numerical model forecasts for many regions in the world including Australia [23], Europe [24] and the United States [25]. For Australia the BoM daily rain gauge analysis dataset [20] is utilised for the validation of 24h-aggregates of satellite rainfall estimates at a 0.25° grid (BoM Centre for Australian Weather and Climate Research (CAWCR) web site [26]). This validation forms the basis of the evaluation we carried out to identify that data product being most useful for the modeling of arbovirus activity in remote tropical areas.

From the great variety of satellite-based precipitation data sources, the Tropical Rainfall Measuring Mission (TRMM) 3B42, the Real-Time TRMM 3B42RT [27] and the Climate Prediction Center (CPC) MORPHed precipitation product (CMORPH) [28] from the National Oceanic and Atmospheric Administration (NOAA) were selected as being potentially useful for our study because of their comparatively high spatial resolution and nearly global coverage.

The TRMM 3B42 product has been available since 1998. It includes merged high quality passive microwave (PMW) and infrared precipitation estimates and Root Mean Square (RMS) precipitation-error estimates adjusted and combined with rain gauge data at a 0.25° by 0.25° resolution, which approximates to a 25 km by 25 km resolution for Northern Australia. The Real-Time TRMM product 3B42RT is computed in near-real time, and constitutes the most timely source of TMPA estimates. While the processing of the 3B42RT dataset requires several simplifications, the 3B42 algorithm is designed to maximise the quality of the estimates [27].

The CMORPH precipitation product incorporates similar data sources to TRMM 3B42. A detailed description of the algorithm can be found in Joyce et al. [28]. CMORPH has been available since December 2002 with a temporal resolution of 30 min and a spatial resolution of approximately 12 km by 15 km. A finer spatial resolution of 8 km by 8 km (at the equator) is obtained via interpolation.

In the work presented, we show how data should be chosen carefully from the vast amount of available data to be most beneficial to a study. We also illustrate how RS rainfall data can be applied to develop models for the prediction of MVEV activity in large and remote areas.

## Methods

### Preparation of serological data

The sentinel chicken data used in this study was limited to the period of 2000 onwards due to the availability of suitable contemporary remotely sensed environmental data. The sentinel chicken seroconversion data were organised in a relational database and the R statistical programming environment [29] was used for further data manipulation. Gaps in the serological data occurred when test results for a given location were not available. To create a dataset appropriate for statistical analysis, periods during which no testing had taken place at a sample site were interpolated where both of the following criteria were satisfied:

1. no seroconversion was detected in a flock when testing recommenced after a testing-free period; and

2. no changes occurred to the flock during the testing-free period.

The remaining gaps (i.e. when seroconversions occurred after a testing-free period) could not be filled and the relevant datasets were further processed by excluding those sample sites with gaps of longer than three months. Datasets with gaps of three months or less were adjusted by setting the date of seroconversion to be at the midpoints of the testing free period. After processing, 20 sentinel chicken flocks remained: 9 locations in the Kimberley and 11 locations in the Pilbara covering the time period from 1 March 2000 until 31 December 2007 (Figure 2).

### Evaluation of TRMM3B42, TRMM 3B42RT and CMORPH

To determine the RS precipitation data product most closely meeting the requirements of this study, evaluation of the TRMM3B42, TRMM 3B42RT and CMORPH was carried out employing a range of statistical measures to quantify different aspects of the satellite precipitation product's performance. Categorical statistics such as frequency bias, probability of detection (POD) or false alarm ratio (FAR) were used to assess each algorithm for its rain occurrence detection skills. As detailed in the Results section below, this led to the selection of TRMM 3B42 for further investigation.

### Processing of TRMM 3B42

Nine years (3288 files) of daily compilations of TRMM 3B42 3-hourly rain estimates for Australia processed within an intercomparison study [30], were provided in four byte real format. The data were processed using ArcGIS^®^and the R statistical programming environment [29]. Geographically projected satellite precipitation images on a daily, monthly and seasonal basis were computed. ArcGIS^® ^focal statistics operations were employed to aggregate the data spatially [31]. Rainfall values of monthly and seasonally accumulated data were extracted at the sentinel chicken flock locations and pixel-based time series were created using R.

### Development of spatio-temporal variables

Based on the knowledge of the ecology of the virus, the principle vector and the principle host, a set of rainfall variables was created and evaluated. These variables represent the rainfall related environmental conditions which influence the dynamics of vector and host populations.

### Seasonal rainfall variables

The seasonal accumulations of TRMM 3B42 focus on the rainfall during different periods of the wet season: December to February (early wet season: *ew*), December to March (early plus high wet season: *ehw*), December to May (complete wet season: *cw*), January to March (high wet season: *hw*), and March to May (late wet season: *lw*) (Table 1). Taking the variation in region-dependent annual rainfall patterns into account, these variables reflect critical epochs of the wet season.

**Table 1:** rainfall variables derived TRMM 3B42 data

Variable	***Description***
***ew***	early wet season (December to February) accumulated rainfall [mm/season/location]
***ehw***	early plus high wet season (December to March) accumulated rainfall [mm/season/location]
***cw***	complete wet season (December to May) accumulated rainfall [mm/season/location]
***hw***	high wet season (January to March) accumulated rainfall [mm/season/location]
***lw***	late wet season (March to May) accumulated rainfall [mm/season/location]
***mr***	the monthly accumulated rainfall [mm/month/location]
***md***	location specific deviation of the monthly accumulated rainfall from the average monthly accumulated rainfall at a location [mm/month/location]
***dba***	location specific accumulated number of days below daily average rainfall in days
***daa***	location specific accumulated number of days above daily average rainfall in days
***mda***	location specific consecutive number of months of positive or negative deviation from the average monthly accumulated rainfall as +/- consecutive number of months

A spatial component was incorporated into the seasonal rainfall variables by using focal mean raster statistics [31] within a 100, 250 and 500 km circular neighbourhood around the sentinel chicken test sites. This was done to allow for larger rainfall catchment areas since local flooding can be caused by runoff from distant rainfall [19,32] and to account for habitat availability for migratory water birds [32,33].

### Monthly time-series

When employing seasonal rainfall variables, modeling was restricted to annual MVEV activity. To enable modeling of virus presence probabilities on a monthly basis, monthly serological data were used together with monthly accumulated rainfall. To account for the temporally lagged nature of processes influencing virus transmission and amplification, e.g. establishment of large vector populations after rainfall events, time-series of monthly accumulated rainfall (*mr*) were compiled from the TRMM data.

### Rainfall anomaly variables

We developed variables based on rainfall anomalies to quantify the magnitude of deviation of the monthly accumulated rainfall from the average monthly accumulated rainfall associated with each month (January till December) at each serological sample site over the study period *(md)*. To take into account anomalies of short- and long-term duration, we used the accumulated number of days below *(dba)*or above *(daa) *the mean daily rainfall associated with each month and the consecutive number of months of positive or negative deviation from the average monthly accumulated rainfall associated with each month and each test site *(mda)*.

### Statistical analysis of spatio-temporal variables

**Figure 3:**
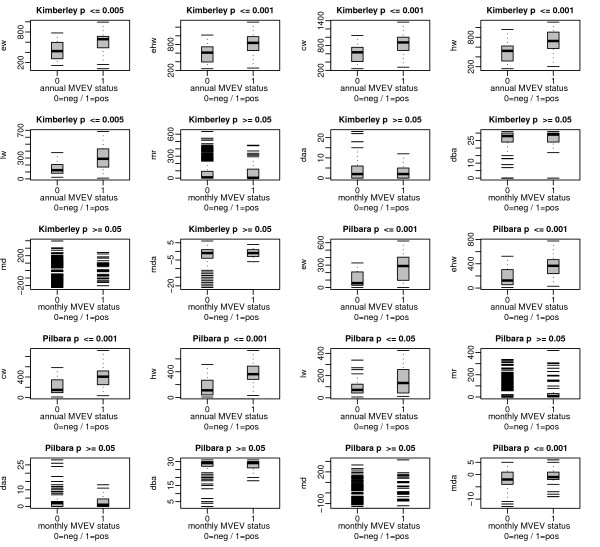
**Correlation of rainfall variables with MVEV status**. Boxplots of seasonal and monthly rainfall variables as presented in Table 1 and annual (October to September) MVEV test site status (0 = MVEV negative; 1 = MVEV positive) and significance of their association determined by Kruskall-Wallis test for significance.

The rainfall variables described above were extracted from the satellite data at the serological sample sites covering the period from 1 March 2000 until 31 December 2007 for bivariate variable screening. A two-tailed Spearman correlation analysis and the Kruskall-Wallis test were used to assess the strength of association between the proposed seasonal/monthly satellite derived rainfall variables and the annual/monthly test site status (positive or negative to MVEV) (Figure 3). The seasonal rainfall variables were correlated with an annual (October to September) status of each test site (Table 2). Using the period from October to September instead of the calendar year as a temporal reference had the advantage of not splitting the wet season during which the main virus activity was expected. Seroconversions of one season can extend into August and September. Monthly time series were tested for their association with monthly MVEV test site status at a series of different time lags.

**Table 2:** Two-tailed Spearman correlation analysis of seasonally and spatially aggregated rainfall variables

Region	***Spatial aggregation***	***None***		***100 km***		***250 km***		*500 km*	
	
	Variable	r	p	r	p	r	p	r	p
**Kimberley**	**ew**	0.3717	≤ 0.01	0.3753	≤ 0.01	0.4261	≤ 0.01	0.4098	≤ 0.01
	**ehw**	0.4225	≤ 0.01	0.4569	≤ 0.01	0.4896	≤ 0.01	0.4732	≤ 0.01
	**cw**	0.4315	≤ 0.01	0.437	≤ 0.01	0.4696	≤ 0.01	0.4551	≤ 0.01
	**hw**	0.4303	≤ 0.01	0.4582	≤ 0.01	0.4979	≤ 0.01	0.4758	≤ 0.01
	**lw**	0.3437	≤ 0.01	0.3142	≤ 0.01	0.3260	< 0.01	0.3172	≤ 0.01

**Pilbara**	**ew**	0.4925	≤ 0.01	0.5117	≤ 0.01	0.5368	≤ 0.01	0.5656	≤ 0.01
	**ehw**	0.4649	≤ 0.01	0.4865	≤ 0.01	0.4829	≤ 0.01	0.4733	≤ 0.01
	**cw**	0.4446	≤ 0.01	0.4709	≤ 0.01	0.4649	≤ 0.01	0.4625	≤ 0.01
	**hw**	0.5538	≤ 0.01	0.5672	≤ 0.01	0.5493	≤ 0.01	0.5466	≤ 0.01
	**lw**	0.2435	≤ 0.05	0.2587	≤ 0.02	0.2139	≤ 0.05	≥ 0.05	≥ 0.05

### Logistic regression modelling

Seasonal and monthly logistic regression models were built for the Kimberley and Pilbara regions. Seasonal models employed a single seasonal explanatory variable at a time. Monthly models were developed using multiple rainfall variables using a backwards stepwise variable selection approach. Odds ratios, adjusted for the effect of other variables in the model and 95% confidence intervals (CI) for MVEV sample site status were calculated. Receiver operating characteristics (ROC) curves [34] were constructed to provide a measure of each model's ability to discriminate between MVEV-positive and MVEV-negative test results.

The logistic regression models developed for the Kimberley and the Pilbara were used to predict MVEV occurrence risk at the sentinel chicken test sites for 2008 and 2009 employing *mr*and *daa *based on the rainfall data of the two years which were not included in the model building process.

## Results

### Investigation of satellite precipitation data

**Figure 4:**
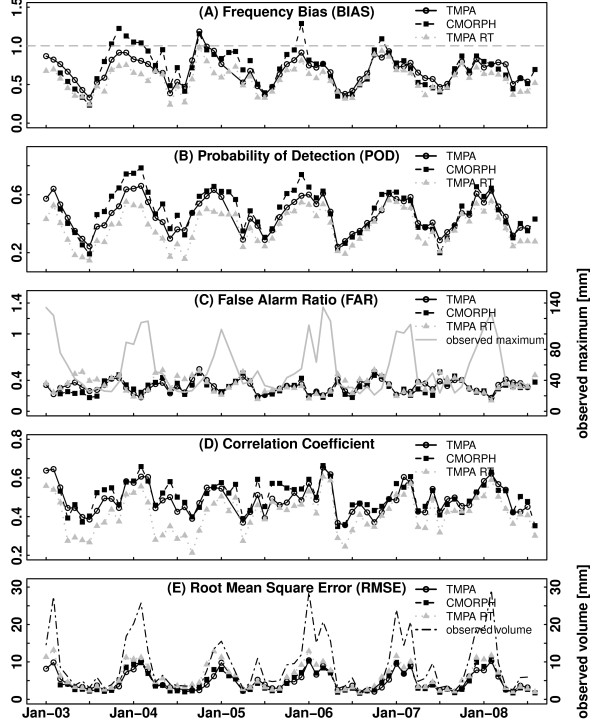
**TRMM 3B42, 3B42RT and CMORPH evaluation statistics**. Statistical comparison of TRMM 3B42, 3B42RT and CMORPH product performance in Australia from 2000 till 2008.

Figure 4 presents summaries of the monthly validation statistics for the TRMM 3B42, CMORPH and TRMM 3B42 RT products from January 2003 to August 2008. All data sets were resampled to a 0.25° by 0.25° (25 km by 25 km at the equator) grid.

The scores were calculated for the whole of Australia and therefore do not consider regional variations. However, information on the performance of the data in different regions, in the form of difference maps displaying deviations of observed rainfall and satellite estimates, can be found on the CAWCR web site [26].

The Frequency Bias or Bias Score (BIAS) (Figure 4A) describes the ability of a satellite precipitation dataset to correctly detect the frequency of rainfall events. A BIAS value of < 1 indicates an underestimate, whilst a BIAS value < 1 shows a tendency of the satellite derived data to overestimate rainfall events. Both the frequency bias scores and the difference maps for TRMM and CMORPH [26] highlight the poorer ability of the CMORPH algorithm. CMORPH data strongly overestimated heavy rainfall as produced by tropical systems, the dominant weather systems during the wet season in north WA, hypothesised to be important drivers of MVEV dynamics. In contrast, TRMM slightly underestimated these events.

The POD (Figure 4B) measures the fraction of observed events that were correctly estimated by the satellite data. The distribution of the POD values for the TRMM and CMORPH products shows consistency with their frequency biases. From the threshold dependent scores (available from the CAWCR web site) it can be seen that precipitation events of higher magnitude are more likely to be detected.

The FAR quantifies the fraction of cases of rainfall estimates in which the event did not occur. Higher FARs seem to be associated with lighter rainfall as illustrated in Figure 4C, where the observed maximum indicates the monthly observed maxima calculated for each location.

The correlation coefficients of the observed data and the satellite precipitation data were similar for the 3B42 and the CMORPH products. Higher correlations were displayed for heavier rainfall (Figure 4D). Both the FAR value distribution and correlation coefficients underline the ability of the satellite precipitation estimates to detect strong rainfall, although the RMS increases with the observed rainfall volume (Figure 4E). The observed volume is derived from the daily rain accumulations at each pixel in the satellite image.

In comparison to the 3B42 and the CMORPH precipitation products, the TMPA real-time data generally showed lower biases, POD scores, and lower correlation coefficients. The FAR score and also the RMS tended to be higher. In summary, the TMPA real-time data did not perform as well as the other data products, which is consistent with the accuracy expectations attributed to these datasets [27]. Based on these results the TRMM 3B42 was determined to be most suitable for this study.

### Significance of rainfall variables

The correlation analysis for spatio-temporal variables, performed separately for the Kimberley and the Pilbara, showed that all spatially non-aggregated seasonal rainfall variables were significantly correlated with annual MVEV status at the test sites in both regions (Table 2). No significant correlations were found between monthly MVEV occurrences and *mr *(Figure 3). The *mda *variable was significantly associated with monthly MVEV presence and absence in the Pilbara (Figure 3) while no significant correlations were found between the rainfall anomaly variables and monthly virus incidence in the Kimberley (Figure 3). For both regions the strongest correlations were observed between *hw *and annual MVEV status at the sample sites, with the spatially aggregated variables showing slightly stronger correlations (Table 2).

### Seasonal logistic regression models

While a logistic regression model using *ehw *performed best for the Kimberley in terms of the model's discriminatory ability (Tables 3 and 4), *ew *was an essential predictor for the presence of the virus in the Pilbara (Tables 5 and 6). For the Kimberley, the model using the 250 km aggregated variable (Table 4) was more significant with a slightly higher odds ratio and higher ROC AUC than a model using smaller (e.g. 100 km) aggregates or non-aggregated data (Table 3).

**Table 3:** Logistic regression employing *ehw *to predict annual MVEV test site status in the Kimberley

Variable	Coefficient (SE)	p	Odds ratio (95%)
**Intercept**	**-2.3410 (0.9322)**	**< 0.05 **^a^	

**ehw (× 100 mm)**	**0.4082 (0.1299)**	**< 0.05 **^a^	**1.50 (1.19-1.99)**^ b^

AIC: 73.911

ROC value: 0.7532609

^a ^Significance

^b ^Interpretation: 100 mm increases in ehw increased the odds of a site annually testing positive to MVEV by a factor of 1.50 (95% CI 1.19 - 1.99)

**Table 4:** Logistic regression employing 250 km aggregated *hw* to predict annual MVEV test site status in the Kimberley

Variable	Coefficient (SE)	p	Odds ratio (95%)
**Intercept**	**-3.6989 (1.1756)**	**< 0.01 **^a^	

***hw *250 km aggregate (× 100 mm)**	**0.7890 (0.2105)**	**< 0.01 **^a^	**2.02 (1.53-3.51)**^b^

AIC: 72.006

ROC: 0.8070652

^a ^Significance

^b^Interpretation: 100 mm increases in 250 km aggregated *hw *increased the odds of a site annually testing positive to MVEV by a factor of 2.02 (95% CI 1.53-3.51)

For the Pilbara, models using both spatially aggregated and non-aggregate seasonal variables were significant (Table 5 and Table 6) while 500 km rainfall aggregates had the strongest associations with annual MVEV detections at the sample sites (Table 6).

**Table 5:** Logistic regression employing *ew *to predict annual MVEV test site status in the Pilbara

Variable	Coefficient (SE)	p	Odds ratio (95%)
**Intercept**	**-1.9873 (0.4741)**	**< 0.01 **^a^	

***ew *(× 100 mm)**	**0.8231 (0.2036)**	**< 0.01 **^a^	**2.28 (1.57-3.52)**^ b^

AIC: 84.457

ROC AUC: 0.79

^a ^Significance

^b^Interpretation: 100 mm increases in *ew *increased the odds of a site annually testing positive to MVEV by a factor of 2.28 (95% CI 1.57 - 3.52)

**Table 6:** Logistic regression employing 500 km aggregated *ew* to predict annual MVEV test site status in the Pilbara

Variable	Coefficient (SE)	p	Odds ratio (95%)
**Intercept**	**-2.8764 (0.6449)**	**< 0.01 **^a^	

***ew *500 km aggregate (× 100 mm)**	**1.4488 (0.3332)**	**< 0.01 **^a^	**4.26 (2.34-8.81)**^b^

AIC: 78.857

ROC AUC: 0.83

^a ^Significance

^b^Interpretation: 100 mm increases in 500 km aggregated *ew *increased the odds of a site annually testing positive to MVEV by a factor of 4.26 (95% CI 2.34 - 8.81)

### Monthly logistic regression models

Tables 7, 8, 9, 10 and 11 show the results of the logistic regression models using time lagged monthly rainfall variables. It was found that *mr *was significantly associated with monthly test site MVEV status in both regions at a lag of three months (Tables 7 and 8). For the Pilbara, associations between *md *and MVEV occurrence were found but resulted in models with comparatively poor discriminatory ability when used as single variables (Table 9).

**Table 7:** Logistic regression employing lagged [lag = 3 months] *mr* to predict monthly MVEV test site status in the Kimberley

Variable	Coefficient (SE)	p	Odds ratio (95%)
**Intercept**	**-2.390 (0.1401)**	**< 0.01 **^a^	

**Lagged *mr *(× 100 mm)**	**0.460 (0.0736)**	**< 0.01 **^a^	**1.58 (1.37-1.83)**^b^

AIC: 583.41

ROC AUC: 0.72

^a ^Significance

^b^Interpretation: 100 mm increases in lagged *mr *increased the odds of a site monthly testing positive to MVEV by a factor of 1.58 (95% CI 1.37 - 1.83)

**Table 8:** Logistic regression employing lagged [lag = 3 months] *mr *to predict monthly MVEV test site status in the Pilbara

Variable	Coefficient (SE)	p	Odds ratio (95%)
**Intercept**	**-3.1939 (0.1660)**	**< 0.01 **^a^	

**Lagged *mr *(× 100 mm)**	**1.3304 (0.1458)**	**< 0.01 **^a^	**3.78 (2.86-5.07)**^ b^

AIC: 452.5

ROC AUC: 0.81

^a ^Significance

^b^Interpretation: 100 mm increases in lagged *mr *increased the odds of a site monthly testing positive to MVEV by a factor of 3.78 (95% CI 2.86 - 5.07)

**Table 9:** Logistic regression employing lagged [lag = 3 months] *md *to predict monthly MVEV test site status in the Pilbara

Variable	Coefficient (SE)	p	Odds ratio (95%)
**Intercept**	**-2.759 (0.139)**	**< 0.01 **^a^	

**Lagged *md *(× 100 mm)**	**1.597 (0.211)**	**< 0.01 **^a^	**4.94 (3.29-7.53)**^ b^

AIC: 479.11

ROC AUC: 0.72

^a ^Significance

^b^Interpretation: 100 mm increases in lagged *md*increased the odds of a site monthly testing positive to MVEV by a factor of 4.94 (95% CI 3.29 - 7.53)

**Table 10:** Logistic regression employing lagged [lag = 2 months] *mr *plus lagged [lag = 3 months] *daa *to predict monthly MVEV test site status in the Kimberley

Variable	Coefficient (SE)	p	Odds ratio (95%)
**Intercept**	**-2.854 (0.183)**	**< 0.01 **^a^	

**Lagged *mr *(× 100 mm)**	**0.3767 (0.083)**	**< 0.01 **^a^	**1.46 (1.24-1.72)**^b^

**Lagged *daa***	**0.124 (0.026)**	**< 0.01 **^a^	**1.13 (1.08-1.19)**^c^

AIC: 557.5

ROC AUC: 0.74

^a ^Significance

^b^Interpretation: after adjusting for the effect of lagged *daa*, 100 mm increases in lagged *mr *rainfall increased the odds of a site monthly testing positive to MVEV by a factor of 1.46 (95% CI 1.24 - 1.72)

^c^Interpretation: after adjusting for the effect of lagged *mr*, single day increases in *daa *increased the odds of a site monthly testing positive to MVEV by a factor of 1.13 (95% CI 1.08 - 1.19)

For both regions, a combination of the different variables including lagged *mr* and lagged *daa*, as presented in Tables 10 and 11, resulted in a model which provided the best fit to the data. This model showed a good discriminatory ability for predicting the presence or absence of MVEV at a given site, as measured by the Akaike Information Criterion (AIC) and ROC AUC (Tables 7, 8, 10 and 11). Logistic regression models using multiple variables enable different effects to be accounted for, the influence of rainfall totals (*mr*) and the influence of the duration of rainfall amounts exceeding the average (*daa*) (Tables 10 and 11).

**Table 11:** Logistic regression employing lagged [lag = 3 months] *mr* plus lagged [lag = 3 months] *daa *to predict monthly MVEV test site status in the Pilbara

Variable	Coefficient (SE)	p	Odds ratio (95%)
**Intercept**	**-3.411 (0.194)**	**< 0.01 **^a^	

**Lagged *mr *(× 100 mm)**	**1.083 (0.167)**	**< 0.01 **^a^	**2.95 (2.14-4.15)**^b^

**Lagged *daa***	**0.096 (0.0350)**	**< 0.01 ^a^**	**1.10 (1.02-1.18)**^c^

AIC: 448.54

ROC AUC: 0.80

^a ^Significance

^b^Interpretation: after adjusting for the effect of lagged *daa*, 100 mm increases in lagged *mr *rainfall increased the odds of a site monthly testing positive to MVEV by a factor of 2.95 (95% CI 2.14 - 4.15)

^c^Interpretation: after adjusting for the effect of lagged *mr*, single day increases in *daa *increased the odds of a site monthly testing positive to MVEV by a factor of 1.10 (95% CI 1.02 - 1.18)

### Monthly prediction of risk MVEV activity

**Figure 5:**
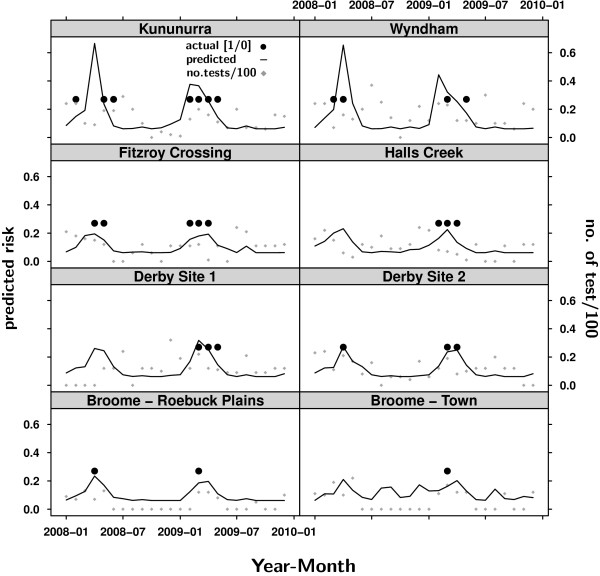
**MVEV status prediction for 2008 in the Kimberley region**. The monthly risk of testing positive to MVEV was predicted for each location in the Kimberley for 2008 and 2009 based on the logistic regression model presented in Table 10. The black solid line shows the predicted risk (between 0.0 and 1.0). The presence of the black dots indicates that a location tested positive during a month, while the grey squares represent the monthly number of tests. For some months for which an increased risk is predicted the number of tests is very low. It is possible that the virus is present in an area without being detected at the sentinel chicken test sites due to a lack of sampling.

**Figure 6:**
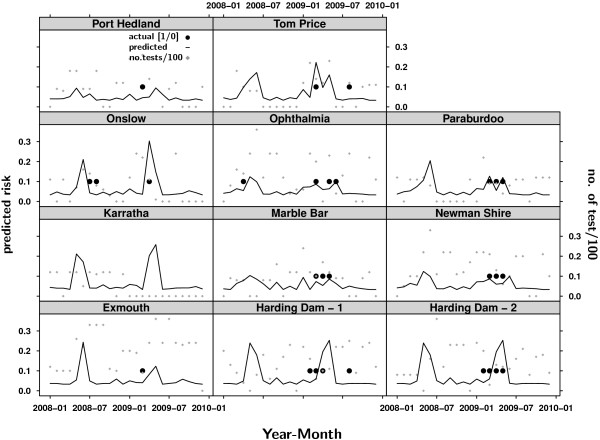
**MVEV status prediction for 2008 and 2009 in the Pilbara region**. The monthly risk of testing positive to MVEV was predicted for each of the Pilbara sentinel chicken locations for 2008 and 2009 using the logistic regression model presented in Table 11. The presence of the black dots indicates that a location tested positive during a month, while the grey squares represent the monthly number of tests. There were very few seroconversions detected in the Pilbara in 2008, which were insufficiently predicted by the model. However the number of samples varied dramatically and there is the possibility of the virus being present according to the modelled risk without being detected. MVEV activity during 2009 was accurately predicted for most sample sites.

Figures 5 and 6 present the modelled MVEV status versus the actual test site status. The predicted risk of MVEV activity was consistent with the actual seroconversions for most flock locations (Figure 5). However, the model overestimated risk for Halls Creek, Derby Site 1 and the Broome sample sites in 2008 (Figure 5). For the Pilbara, risk at the sample sites was predicted accurately in many cases (Figure 6). For some sites the prediction showed slight temporal offsets to the detection of seroconverions in the sentinel chickens. Overestimates of risk are visible for 2008. The late seroconversion in the 2009 season at Tom Price was not predicted by the model. The overall accuracy as measured by the ROC AUC was 0.93 for the Kimberley and 0.75 for the Pilbara, respectively.

## Discussion

### Selection of satellite precipitation data

TRMM 3B42 data provide us with the most accurate and spatially regular available information for the entire study area. The verification results, particularly the difference maps [26], showed that TRMM 3B42 data performed well for stronger rainfall events which are associated with the dominant weather systems of the northern parts of WA during the wet season and which are likely to create surface water areas that persist for sufficient time to enable vector breeding, as well as to attract large numbers of hosts. CMORPH was rejected for this study as it showed pronounced overestimates in the Kimberley region, and data were not available before December 2002. TMPA real-time data, although less accurate than 3B42 data, may be useful for real-time applications, especially for predictive modeling, since they are available within 24 hours whereas 3B42 data are published approximately one month from the date of recording.

### The effect of rainfall on MVEV test site status

Logistic regression models employing rainfall based predictor variables had a greater discriminatory ability to predict MVEV activity in the Pilbara (ROC AUC: 0.72 - 0.83) (Tables 5, 6, 8, 9 and 11) than the Kimberley (ROC AUC: 0.70 - 0.79) (Tables 3, 4, 7 and 10), and also showed consistently larger odds ratios, suggesting that the amount of rainfall has a stronger effect on the MVEV status in the Pilbara than in the Kimberley, both monthly and seasonally. This may be linked to the ecologically different backgrounds the two regions provide for virus activation and maintenance. In terms of rainfall, temperature regimes and hydrological landscape characteristics [26,35,36], the Kimberley generally provides more favourable conditions for vectors and hosts of MVEV than the Pilbara. Hence the virus is enzootic (constantly present) in the Kimberley, irrespective of the intensity of the wet season, which may be one explanation for a less pronounced statistical association between rainfall and MVEV occurrence in the Kimberley. However, other environmental factors not considered in this study influence MVEV ecology and may lead to above average activity of MVEV in the Kimberley region during some years.

### Seasonal variables

The seasonal variables *hw *and the *ehw *for the Kimberley and *ew *for the Pilbara were found to be more important overall than the other seasonal variables (Table 2). This can be related to the climatic regimes of the two regions. Large amounts of rain in the Kimberley between December and March contribute to the maintenance of large wetland habitats during these months and for longer time periods. The usually lower rainfall in the Pilbara is spatially more heterogeneous. Inundated areas in the Pilbara tend to be smaller and not as long-lasting as in the Kimberley. In the Pilbara rain falls between December and July but reaches comparatively high values in January, February and March suggesting that *hw*should be significant. However, employing *ew *in the logistic regression analysis (Tables 5 and 6) led to the model providing the best fit to the data, suggesting that additional cross-regional or cross-seasonal rainfall, or non-rainfall related factors are also involved.

### Spatially aggregated seasonal variables

Spatially aggregated seasonal variables (within a 100, 250 and 500 km circular neighbourhood of each flock location) were utilised to consider larger areas around the serological sample sites. This was done to account for often widespread rainfall in the study area, potentially leading to the creation of widely dispersed or extensive wetlands, as well as for the ability of flood waters to move over hundreds of kilometres into large drainage basins [19]. Spatially aggregated variables also reflect the responses of water birds to wetland distribution on a local, catchment or beyond catchment scale. Water birds are able to exploit widely dispersed wetland habitats [32]. While all focal variables (100, 250 and 500 km) were significantly related to annual MVEV activity in both regions, two of the variables seemed to be more crucial for virus activity, the 250 km variable for the Kimberley and the 500 km variable for the Pilbara region, respectively (Tables 2, 4 and 6). This again, may be related to the different climate and land forms dominating the two regions. While relatively large quantities of rainfall together with extensive water absorbing environments are found in the Kimberley, the land forms of the Pilbara naturally restrict the spatial extent of flooding caused by already more ephemeral precipitation events and a larger overall area may be required for the creation of suitable mosquito vector and host habitat to the same extent.

### Time-lagged monthly rainfall variables

The lack of association between monthly MVEV activity and monthly rainfall variables (Figure 3) is explained by the temporal characteristics of the ecology of the virus. Time lags of two to three months were significantly associated with virus detection in sentinel chicken sera in this study (Tables 7, 8, 9, 10 and 11). There may be several factors responsible for the significant relationship between lagged rainfall and MVEV activity. When wetlands fill with water due to heavy rainfall, two main ecological processes are initiated that facilitate virus amplification in the bird-mosquito-bird cycle; the assembly of water bird flocks, and the establishment of mosquito vector populations. Certain amounts of time are required for each of these ecological processes to occur. Exact times are still poorly understood and strongly depend on the environmental circumstances as well as cross-linked sub-processes such as competition. The response of most bird species to flood related resource pulses is likely to be within days or weeks [37]. Whelan et al. [16] showed increased numbers of the principal vector *Cx. annulirostris *two to three weeks after heavy rainfall and widespread flooding in the Northern Territory. *Cx. annulirostris *requires certain environmental conditions for breeding, such as shallow water with emerging vegetation [3] which in turn depend on rainfall quantities. These conditions are highly variable over space and time. Therefore, times for the development of large vector populations may vary in response to environmental conditions (e.g. rainfall, humidity and temperature) at different locations. For example if less mosquito breeding habitat is available it might take longer for large mosquito populations to establish [38]. Vector breeding and survival is also strongly related to temperature and the presence of predators. A study by McDonald and Buchanan [39], carried out under controlled environmental conditions, showed that *Cx. annulirostris *mosquitoes exploited shallow water pools with emerging vegetation within hours of their formation. Larvae have been found within two to three days [40]. The development from larvae to adult mosquitoes may take from one week to 25 days [40]. Russell [38] showed that times from blood feeding to oviposition in *Cx. annulirostris *ranged from four to twelve days. However these times can only be used as an approximate guide and are expected to be different depending on time and location. Furthermore, the time required for development of large vector populations depends on whether and how many vectors are permanently present during the dry season, either in permanent smaller local water bodies (e.g. permanent pools, sewage ponds or irrigated areas) or as long-lived adult mosquitoes, or whether the vector needs to be re-introduced into an area. Furthermore, vector survival, and perhaps virus survival, is possible in the form of desiccation resistant eggs of other species such as *Aedes normanensis *[41].

The two and three-months time lags suggest that more than one cycle of amplification, as illustrated by Konno [42] for Japanese encephalitis virus, is needed for the virus to become established in an area after an initial environmental signal, such as a rainfall event. Once host and vector populations have established it may take several cycles for the virus to amplify to detectable levels. After the host has been infected by an adult female mosquito, viraemia develops within one to two days and may last for three to five days [43,44]. Only during this time can other mosquitoes blood-feeding on the host become infected, before the host's immune response clears the virus from circulation.

Virus amplification is influenced by a number of factors such as whether or not the virus is already present in an area, the abundance and population structures of competent vectors and susceptible hosts as well as the development of viraemia in the host, depending on the host species and age [43]. The presence of large proportions of immune hosts in a population one or more years after a season with intense virus activity might limit the amplification cycle as these hosts will not develop viraemia [45]. In addition there might be differences in vector competence within the same species depending on the origin of the vector [46].

### Prediction

The predictions performed for 1 January 2008 until 31 December 2009 (Figures 5 and 6) show a good ability of the models to predict the risk of MVEV presence. In some cases the models overestimated the risk of MVEV activity (Figures 5 and 6). In other cases temporal discrepancies existed between the predicted risk of activity and the actual detection of seroconversions. Explanations for overestimates and temporal discrepancies relate to the sampling. First, the virus may be present in an area without necessarily being detected in the sentinel chickens at the sample site. Second, the serological sampling was not always carried out regularly and the number of chickens tested varied between the test dates and locations, such that virus activity may therefore have occurred without being detected or being detected later, when sampling recommenced after a testing-free period as shown in Figure 6 for Tom Price in 2009. A potentially beneficial area of future research would be to sample more intensively in areas where MVEV risk appears to have been overestimated (e.g. Halls Creek and Derby Site 1) to definitively rule out the presence of viral activity.

The model results imply that due to the environmental conditions at a specific location and time, the risk of virus activity may be increased. Other non-rainfall environmental conditions were not considered in this study, but are known to influence MVEV activity. Incorporation of these factors might increase model accuracy.

## Conclusions

Satellite based precipitation data represent a useful data source for the analysis of spatio-temporal rainfall patterns associated with arbovirus activity, especially for larger (regional to continental) areas. Unlike point measurements, or spatially interpolated surfaces based thereon, which are expected to be inaccurate in areas where ground measurements are sparse, satellite based precipitation estimates are spatially regular and cover remote and difficult to access areas.

The data are not only capable of spatially coherent mapping of MVEV risk, but also enable the development of spatio-temporal rainfall variables accounting for the surrounding environment of a location of interest as well as for the temporally lagged nature of ecological processes responding to changing environmental conditions. This is important, since the virus, mosquito vectors and animal hosts are influenced by environmental factors, which are often extremely spatially and temporally variable. Furthermore, the utilisation of the satellite precipitation data enables the consideration of regional and super-regional scale processes such as the assembly of water birds, which are known to move across large areas depending on habitat availability, and also of the influence of rainfall in larger catchment areas.

TRMM 3B42 data are currently available one month delayed, which given the significance of the 3-monthly lagged rainfall variables, still enables on-time risk mapping and is therefore capable of providing early warning.

The analysis and modelling of MVEV dynamics in response to environmental variability are limited by the scarcity of ground truth information on mosquito vector and host abundance required for verification of the identified relationships. The likely involvement of other mosquito vector and host species [3,44] with different ecologies that may be less controlled by the variables incorporated into the models developed in this study are also confounding factors.

Nevertheless, based on the knowledge of the ecology of the principle vector and hosts, it was possible to develop key rainfall variables and to employ these in the prediction of the risk of MVEV activity at the serological sample sites. The results shown here are consistent with a range of studies [40,47,48], illustrating that other non-rainfall related environmental variables such as temperature, humidity, wind and vegetation are also likely to be important in MVEV ecology. Work on the incorporation of these additional variables into new models is in progress.

In the future the prediction models presented in this paper should be further validated by employing spatially denser ground truth information on virus activity collected in selected sample areas which are easier to access. This would then allow RS rainfall data to be routinely used as a tool to predict the spatial distribution MVEV activity which, in turn, should provide the general public with important information about disease transmission risk. However the acquisition of ground truth data on virus presence/absence in an area is expensive and might therefore be restricted.

The frame work of data and methods described in this paper may be valuable for similar applications for other medically important arboviruses with complex ecologies such as WNV and SLEV.

## Competing interests

The authors declare that they have no competing interests.

## Authors' contributions

GS designed the study and acquired the RS data. She processed the RS and sentinel chicken data, derived the environmental variables, carried out the statistical analysis and drafted the manuscript. EEE evaluated and validated the satellite precipitation data on which this research is based and participated in drafting the manuscript. MAS assisted in the construction of the statistical analysis framework as well as in drafting the manuscript. RJC significantly contributed to design the study and to the preparation the manuscript. CAJ participated in conceptualisation of the study design, the interpretation of the analyses and the preparation of the manuscript. The sentinel chicken data were acquired by the Western Australian Arbovirus Surveillance and Research Program lead by CAJ. All authors read and approved the final manuscript.
